# Phantom-based experimental validation of fast virtual deployment of self-expandable stents for cerebral aneurysms

**DOI:** 10.1186/s12938-016-0250-6

**Published:** 2016-12-28

**Authors:** Qianqian Zhang, Zhuangyuan Meng, Ying Zhang, Kai Yao, Jian Liu, Yisen Zhang, Linkai Jing, Xinjian Yang, Nikhil Paliwal, Hui Meng, Shengzhang Wang

**Affiliations:** 1 Beijing Neurosurgical Institute, Beijing Tiantan Hospital, Capital Medical University, Beijing, China; 2 Department of Mechanics and Engineering Science, Fudan University, 220 Handan Rd., Yangpu District, Shanghai, 200433 China; 3 Toshiba Stroke and Vascular Research Center, University at Buffalo, The State University of New York, Buffalo, NY USA

## Abstract

**Background:**

Endovascular intervention using a stent is a mainstream treatment for cerebral aneurysms. To assess the effect of intervention strategies on aneurysm hemodynamics, we have developed a fast virtual stenting (FVS) technique to simulate stent deployment in patient-specific aneurysms. However, quantitative validation of the FVS against experimental data has not been fully addressed. In this study, we performed in vitro analysis of a patient-specific model to illustrate the realism and usability of this novel FVS technique.

**Methods:**

We selected a patient-specific aneurysm and reproduced it in a manufactured realistic aneurismal phantom. Three numerical simulation models of the aneurysm with an Enterprise stent were constructed. Three models were constructed to obtain the stented aneurysms: a physical phantom scanned by micro-CT, fast virtual stenting technique and finite element method. The flow in the three models was simulated using a computational fluid dynamics software package, and the hemodynamics parameters for the three models were calculated and analyzed.

**Results:**

The computational hemodynamics in the patient-specific aneurysm of the three models resembled the very well. A qualitative comparison revealed high similarity in the wall shear stress, streamline, and velocity plane among the three different methods. Quantitative comparisons revealed that the difference ratios of the hemodynamic parameters were less than 10%, with the difference ratios for area average of wall shear stress in the aneurysm being very low.

**Conclusions:**

In conclusion, the results of the computational hemodynamics indicate that FVS is suitable for evaluation of the hemodynamic factors that affect treatment outcomes.

## Background

Cerebral aneurysms are pathological dilatations of cerebral vessels due to a weakening of the layers of the vessel wall. The rupture of cerebral aneurysms has catastrophic consequences, with high mortality and morbidity rates, as well as high health care costs. With the rapid improvement of medical imaging and neurovascular techniques, the treatment options for intracranial aneurysm are increasingly promising. The deployment of self-expandable stents typically plays a key role in aneurysm treatment options. Intracranial stenting is often used to provide a scaffold aimed at holding coils inside the aneurysm sac (stent-assisted coiling). It is assumed that such stents will also cause a reduction to the intra-aneurysmal flow, with the extent depending on the design of the stent [1]. However, aneurysmal interventions are complicated by the lack of knowledge on the specific conditions of each stent deployment.

In recent years, virtual stenting methods have been developed and applied to support clinicians in the planning of aneurysmal stenting procedures. To the best of our knowledge, the virtual stenting methods currently in use can be categorized into two types, according to the mechanism employed; these are the finite-element-method (FEM), and the fast virtual stenting (FVS) technique. Ding Ma et al. applied the finite element method to simulate the mechanical deployment of braided stents in patient-specific aneurysms [2]. Many other studies on virtual stenting using FEM have now been published [3–5]; these include qualitative and quantitative analysis, and FEM is now well accepted, although the computational time costs are high. The other published technique is FVS, which provides an estimation of the configuration of intracranial stents [6]. Ding Ma [7] proposed an enhanced fast virtual stenting method applied with a push–pull technique; this had the unique capability of accurately representing stent interventions in silicon models. However, quantitative validation of FVS analysis against experimental data has not been fully addressed. It is unclear if this method can reproduce the highly variable deployment configurations of patient-specific cases, and thereby enable accurate analysis of post-treatment hemodynamics. These analyses are vital to evaluate variations in treatment outcomes.

With the advances in endovascular treatments over the years, many studies on aneurysms have been performed, and many authors have reported on the hemodynamic factors related to aneurysms [8]. Knowledge of these hemodynamic characteristics is most certainly helpful for improving the understanding of the detailed procedures associated with aneurismal growth, evolution, and rupture. Computational fluid dynamics (CFD) is a robust non-invasive and promising tool that has been applied successfully to the study of hemodynamic changes in aneurysms. In order to illustrate the realism and usability of the novel FVS method, we performed in vitro studies of a patient-specific model with quantitative CFD analysis. We compared the experiments and simulations to check the quality of the numerical predictions, and to ensure they provided important information for clinical applications.

## Methods

### Patient specific aneurysm model

This study was approved by the ethics committee of Beijing Tiantan Hospital and informed consent was obtained from the study patient. A wide-neck internal carotid artery (ICA) aneurysm (width 12.27 mm, height 9.35 mm) was selected from digital subtraction angiography (DSA) image databases collected by our Interventional Neuroradiology Laboratory (Beijing Tiantan Hospital, China). The images of the patient-specific aneurysm were used to create a 3D reconstruction, which was then used to create a stereolithography format file of the initial geometry for the CFD simulation. The maximum diameters of the parent vessel and outlet branches at the trifurcation were 4.68, 3.01 mm (middle cerebral artery), and 2.64 mm (anterior cerebral artery).

**Fig. 1:**
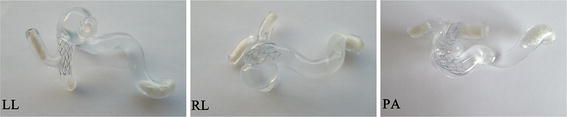
Photographs of the patient-specific aneurismal phantom visualized by three views we defined. *LL* left lateral; *RL* right lateral; *PA* posterior–anterior

A patient-specific aneurismal phantom was fabricated using stereolithography file and prototyping techniques. This model should reflect the real human anatomy, and was therefore considered to be well suited to our present study. Three views of the phantom with a deployed Enterprise stent are shown in Fig. 1: left lateral (LL), right lateral (RL), and posterior–anterior (PA).

### Deployment of the stent in the three simulated methods

The modeling workflow of the novel FVS deployment incorporated several simplifications [9]. First, the aneurysm geometry was isolated from the 3D patient-specific aneurism model using Geomagic Studio (Raindrop Geomagic, Research Triangle Park, NC, USA), and the centerline of the parent vessel was extracted using Mimics 10.01 (Materialise, Leuven, Belgium). After generation of the vessel centerline, a simplex mesh was initialized and treated as a deformable simplex model that was expanded using MATLAB 2013 (MathWorks, Natick, MA, USA). The deployment stopped when the simplex mesh achieved good apposition with the parent-vessel wall, thus giving the optimal deployed simplex mesh. The Enterprise stent patterns were then mapped onto the deployed simplex mesh and the wire was then swept, with the aim of obtaining the 3D structure of the stent.

In the patient-specific physical phantom model, an Enterprise stent was released into the parent artery of the saccular aneurysm by an experienced neurosurgeon. To conduct a CFD analysis of this experimental phantom, a micro-cone-beam computer tomography (micro-CT) system with a resolution of 10 µm was employed to scan the physical phantom and reconstruct the deployed Enterprise stent. The deployed stent was then reconstructed and a stereolithography format file was generated. An FEM deployment was also performed in accordance with the methods described in a previous study [10].

### CFD simulation and hemodynamic analysis

Computational fluid dynamic analysis was performed for three scenarios: the stented aneurysm model from the realistic deployment, the FVS deployment, and the FEM deployment. The flow-governing Navier–Stokes equations were solved to second-order accuracy using ANSYS CFX 14.0 (ANSYS inc., Canonsburg, PA, USA). Velocity and pressure fields were computed with the common assumptions of incompressibility, Newtonian flow, rigid walls, and steady-state conditions. The density and viscosity of blood flow were input as 1050 kg/m^3^ and 4.0 cP respectively [11]. The mean flow rate at the inlet was set to maintain a wall shear stress of 1.5 Pa at the inlet, and the outlet pressure was set to 0 Pa. Several hemodynamic parameters, such as streamline, wall shear stress (WSS), area average of WSS on aneurysm, and average velocity inside the aneurysm, were calculated to validate the FVS according to CFD.

## Results

**Fig. 2:**
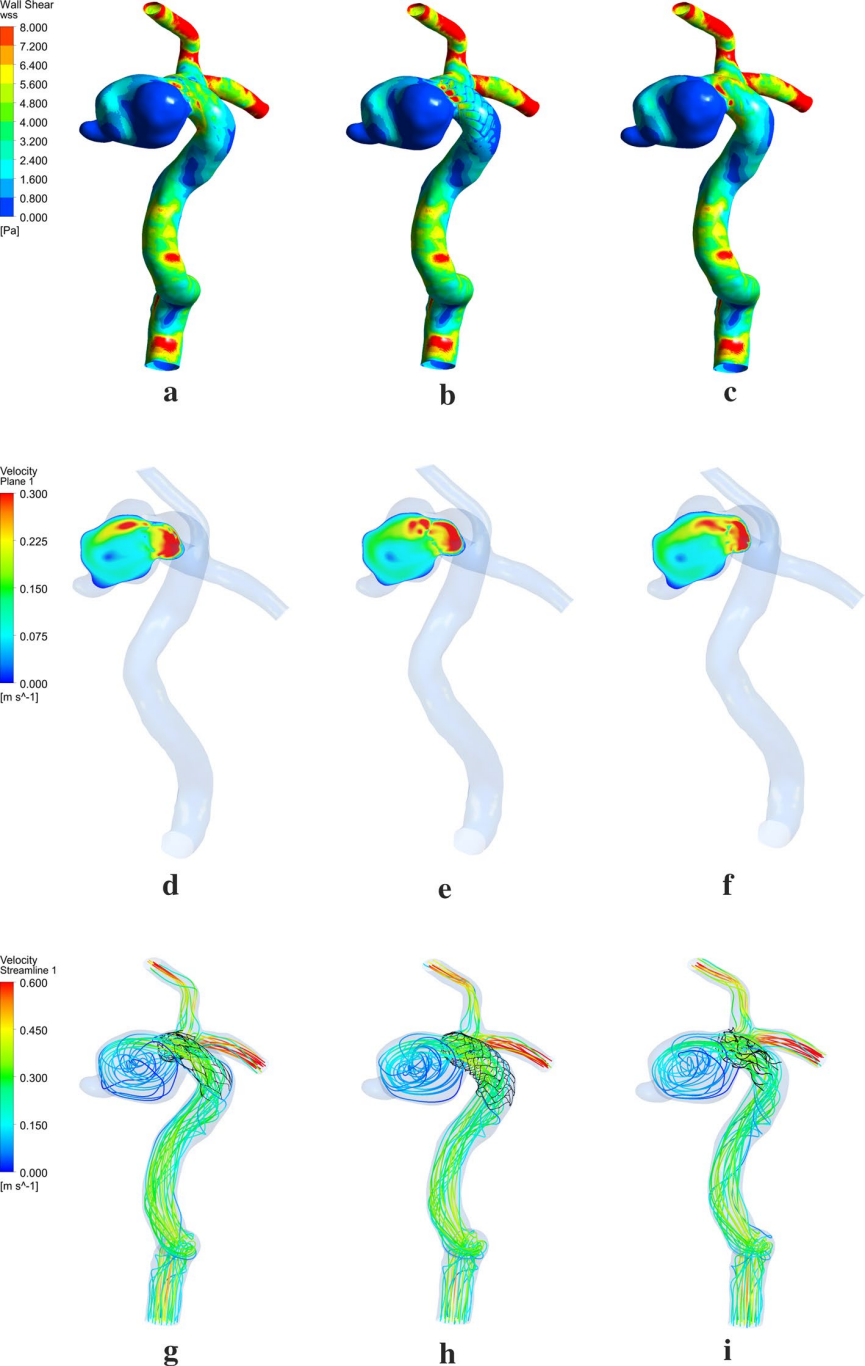
Demonstration of wall shear stress **a**–**c** velocity plane, **d**–**f** and streamline, **g**–**i** in three stenting models created by different methods

As CFD models have been well validated, we used the CFD results for comparison purposes and to validate our FVS method. Qualitative visual comparisons between the FVS, FEM, and the realistic deployment of the Enterprise stent are shown in Fig. 2. The WSS, streamline, and velocity plane in the three different methods are all similar. Examination of WSS (Fig. 2a–c) shows that areas with distinct low WSS distributions are consistently found in the aneurysm, while areas with slightly higher WSS distributions are observed around the aneurysm neck. The velocity magnitudes within the aneurismal plane showed similar trends in all three models (Fig. 2d–f), with a larger area percentage of high velocity distributions in the FVS (Fig. 2e) and FEM (Fig. 2f) models. As was the case for the velocity magnitudes, the velocity streamlines of FVS and FEM were a little higher in the vortex than in the realistic phantom model (Fig. 2h–g).

For the quantitative comparison, the hemodynamic parameter, including area average of WSS on aneurysm, area average of WSS on parent vessel, and average velocity inside the aneurysm, were accurately calculated. The data are presented in Table 1 and the difference ratios of the parameters are presented in Table 2. We took the value of the hemodynamic parameters in the realistic phantom model as the reference, so that the difference ratios of the hemodynamic parameters between the phantom model stenting deployment and the FVS, and between the phantom model stenting deployment and the FEM, were calculated. All of the difference ratios were less than 10%, with the difference ratios for the area average of WSS on the aneurysm (Table 2) being very small. The average velocity within the aneurysm with the FVS method had a greater magnitude than with the realistic phantom, with the difference ratio between the two being approximately 10%. In contrast, the difference ratio for the average velocity within the aneurysm was very similar between the FVS and FEM methods. The area average of the WSS in the aneurysm (Pa) with the FVS method had a difference ratio of 1.26% from the realistic phantom, which was smaller than the 1.95% calculated between the FEM method and the realistic phantom. However, the difference ratio of the area average of WSS on the parent vessel (Pa) was slightly larger with FVS than with FEM (4.97 vs 2.83% respectively). There is no doubt that both our FVS method and the FEM method had differentiations with the method of realistic in some degree.

**Table 1:** Hemodynamic parameters of the parent vessel in the three different methods

	Average of velocity inside aneurismal sac (m/s)	Area average of WSS on the aneurysm (Pa)	Area average of WSS on the parent vessel (Pa)
Realistic (X)	0.0829	0.8317	4.1118
FVS (Y)	0.0908	0.8212	3.9075
FEM (Z)	0.0905	0.8155	4.2283

*X* corresponding hemodynamic parameters in realistic phantom deployment of Enterprise stent; *Y* corresponding hemodynamic parameters in FVS simulation of Enterprise stent; *Z* corresponding hemodynamic parameters in FEM simulation of Enterprise stent; *WSS* wall shear stress

**Table 2:** Percentage differences in the hemodynamic parameters of the parent vessel between the three different methods

	D1 (%)	D2 (%)
Average of velocity on aneurysm (m/s)	9.52	9.17
Area average of WSS on aneurysm (Pa)	1.26	1.95
Area average of WSS on parent vessel (Pa)	4.97	2.83

D1 = |(Y-X)|/X; D2 = |(Z-X)|/X; *X*, *Y*, and *Z* represent corresponding hemodynamic parameters from the realistic phantom, FVS simulation, and FEM simulation respectively. *WSS* wall shear stress

## Discussion

The present study evaluated the performance of the FVS method in stenting procedures for cerebral aneurysms. From a hemodynamic viewpoint, there were slight differences between the results of the FVS and those of the realistic phantom stenting deployment and FEM. As can be seen in Fig. 2, with the FVS procedure, the shape and geometry of the stent and the strut apposition to the vessel wall are distinctly different to the other two methods. Both the realistic phantom stenting deployment and the FEM showed malapposition to the parent-vessel wall, which may have been caused by the regions of curved vessel. It can also be observed that the shape of the stent using FEM was more similar to that of the realistic phantom. However, the difference ratios of the hemodynamic parameters calculated by CFD simulation are relatively small, so we therefore consider the FVS method to be acceptable. Additionally, we compared results against a physical phantom of the patient-specific cerebral aneurysm, and consider this to be an advantage of this research in comparison with aneurysm studies based only on idealized or simplified aneurysm models. The results of this study indicate that the combination of FVS with CFD should ultimately become a useful method for assessment of the best possible treatment options, and evaluation of the post-treatment prognosis.

The stent length, angle between the struts, and strut width, length, and diameter, should be taken into account, as these stent characteristics are sufficient to influence the hemodynamics and the treatment outcome. As is the case in most CFD simulations, we made the assumption of a rigid vessel wall, which allowed us to use Newtonian blood properties, and therefore, some variables such as vascular resistance, could not be quantitatively evaluated. Pulsatile blood flow, especially patient-specific flow velocity, could be applied to better quantify the accuracy of such simulations. There is no doubt that improved virtual modeling of stenting and coiling is urgently needed, as it can describe in a realistic manner the relevant hemodynamic factors and flow modifications. Improvements to FVS require more collaboration between engineers and clinicians.

## Conclusions

In this work we performed numerical validation of FVS, a novel methodology for virtual stent release. The results indicate that FVS has the ability to perform virtual stenting, and that it will allow the evaluation of the flow dynamics and hemodynamics that would occur after the stenting of cerebral aneurysms. Accordingly, we are confident that our FVS technique provides an effective model of a stented aneurysm, and in combination with CFD, allows study of the ensuing hemodynamics. The method does suffer from some limitations; however, the results could still be validated.

In conclusion, the FVS technique, which is easier to automatically implement than FEM, may be a good approach to model the deployment of stents. If the clinicians are directly able to understand the FVS without the presence of an engineering expert, this approach should be strongly recommended for virtual stenting in endovascular interventional planning.
